# Evaluating the Residual Nitrite Concentrations of Bacon in the United Kingdom

**DOI:** 10.3390/foods9070916

**Published:** 2020-07-11

**Authors:** William Crowe, Christopher T Elliott, Brian D Green

**Affiliations:** Institute of Global Food Security, School of Biological Sciences, Queens University Belfast, Belfast BT9 5DL, UK; w.crowe@qub.ac.uk (W.C.); chris.elliott@qub.ac.uk (C.T.E.)

**Keywords:** nitrite, bacon, processed meat

## Abstract

The preservative sodium nitrite is added to processed meat with the intention of preventing the growth of *Clostridium botulinum,* but this also influences product flavour and colour. The World Health Organisation has declared nitrites to be ‘probably carcinogenic’. Use is permitted by the European Union but its addition is limited to 100 mg/kg in all processed meat, except bacon, which is limited to 175 mg/kg. At present, there is no independent peer-reviewed literature assessing the residual nitrite levels in bacon in the United Kingdom. Furthermore, this is the largest study of residual nitrite concentrations in bacon that has ever been conducted. A total of 89 different commercially available bacon samples were collected, and analysed using flow injection analysis to determine their residual nitrite content. The mean residual nitrite concentration for all bacon samples was 10.80 mg/kg. Residual nitrite levels did not differ between smoked and unsmoked bacon. Middle cut bacon (26.00 mg/kg) had significantly higher residual nitrite concentrations than back bacon (8.87 mg/kg; *p* = 0.027), and medallion bacon (4.47 mg/kg; *p* = 0.008). This study shows that there is large variation in the mean residual nitrite levels of bacon sold in the UK and all the reported values are within current regulatory limits. Despite this, it appears that many manufacturers could decrease the amount that they are currently using in their products.

## 1. Introduction

Nitrite salts are curing agents added to processed meat with the aim of enhancing shelf life, flavour, and colour. Commonly used forms of nitrite salts include sodium nitrite and potassium nitrite. Nitrite salts are effective antimicrobial agents that elicit their effects by decreasing water potential, delaying oxidative rancidity, and subsequently preventing the growth of bacteria. Manufacturers typically use nitrite salts to prevent the growth of *Clostridium botulinum* [1]. *Clostridium botulinum* is a rod-shaped anaerobic bacteria that produces botulinum toxin, which is responsible for causing the neuroparalytic condition botulism. In severe cases, botulism can lead to respiratory failure and death. Botulism is rare in Europe—the incidence rate has ranged between 85 and 124 annually in the past 10 years [2]. Nitrite also influences the colour of meat. Oxymyoglobin is responsible for the red/pink colour of meat. The loss of an electron from oxymyoglobin leads to the formation of metmyoglobin, subsequently turning the meat brown. The meat colour change is temporary, given that many things can influence this oxidation reaction, such as the presence of bacteria, aging and cooking. Nitric oxide (NO) arising from sodium nitrite combines with myoglobin in the presence of deoxymyoglobin to form the heat-stable NO myoglobin. The NO acts as a substitute for oxygen, contributing an increasing pink colour to the meat [3], which is a desirable trait for consumers [4]. Unreacted nitrite within meat is known as residual nitrite, and this portion is readily measurable. Some manufacturers substitute nitrite for vegetable extracts that contain nitrate to cure meats, which ultimately results in the generation of nitrite [5], and these products are marketed as natural bacon. Uncured meats refer to meat where no nitrite or equivalent has been added. Consumers indicate a preference for bacon with nitrite added, and score it higher for colour acceptability, flavour, and texture [6]. Concerns have been raised with regard to the safety of natural bacon, with studies showing higher levels of bacterial growth present on their surface [7].

Processed meats have been classified as a group 1 carcinogen by the International Agency for Research on Cancer (IARC) [8], and they define processed meat as any meat that has been altered through salting, curing, smoking or other processes, with the aim of preserving or improving the flavour of the meat. It is reported that the daily consumption of 50 g of processed meat, which is approximately two rashers of bacon, increases the risk of colorectal cancer (CRC) by 18% [8]. It is unclear what constituent(s) present in processed meat are responsible for the cancer-promoting effects. Leading candidates include nitrites, polycyclic aromatic hydrocarbons (PAHs), haloacetic acids (HAAs), haem iron, and saturated fats. Nitrites have emerged as the foremost contender due to their ability to generate N-nitroso compounds (NOCs), some of which are known to be carcinogenic [9]. Nitrite is classed as a group 2A carcinogen and described as probably carcinogenic [10]. A European Parliament and Council Directive has restricted the addition of sodium nitrite in processed meat to 150 mg/kg, and the residual amount must be below 100 mg/kg. In cured bacon, the residual amount must be below 175 mg/kg (EC 95/2/EC) [11]. The joint Food and Agriculture Organisation of the United Nations/World Health Organisation (FAO/WHO) expert committee on food additives (JECFA) has agreed an acceptable daily intake (ADI) of nitrite to be 0.07 mg/kg bodyweight [1]. This dosage was calculated whilst considering carcinogenicity and the development of methaemoglobin [1]. The ADI is the quantity of a compound that can be consumed every day over a lifetime without conferring any health risk.

According to EU Regulation No. 1169/2011, all products with nitrite added must list this on the ingredient list, either explicitly as sodium/potassium nitrite or stating the E number (E250/E249). However, the quantity of nitrite does not need to be specified on the label. At present, there is no independent scientific literature determining the residual nitrite concentrations found in bacon sold in the UK. The aim of this investigation was to measure the mean residual nitrite concentration of bacon samples available in the UK, and the secondary aim was to compare the residual nitrite concentrations of bacon of different types and cuts.

## 2. Materials and Methods 

### 2.1. Samples

A total of 89 bacon samples were purchased from the shelves of 4 large UK supermarket chains (Asda, Sainsbury’s, Tesco and Lidl). Samples were all purchased on the same day and stored in refrigeration at 4 °C until shipment, which occurred on the same day of purchase. Samples were removed from original packaging and placed in transparent plastic bags, anonymised and coded. Samples were shipped in Styrofoam containers lined with ice packs to Eurofins scientific (Wolverhampton, England). Samples were non-discriminatorily purchased based on their availability, which represents consumer purchasing trends. Commercially available bacon samples included back (*n* = 52), streaky (*n* = 13), middle (*n* = 6), medallions (*n* = 15), and diced (*n* = 3). 

### 2.2. Determination of Residual Nitrite Levels

The proximal analysis values self-reported by manufacturers on product labels were recorded in a database and included in statistical analysis (Table 1). Five grams of each bacon sample was weighed to the nearest 0.1 mg and triturated for 5 min in individual mortars. Distilled water was added at a volume of 40 mL and the mixture was added to a water bath at a temperature of 80 °C for 120 min. The liquid was passed through a 0.45 m Whatman syringe filter twice before analysis. All samples were analysed for nitrite concentrations using the flow injection analysis (FIA) method [12]. A nitrite standard at a concentration of 1000 µg/mL^−1^ was prepared by adding 150 mg of sodium nitrite (Merck, Darmstadt, Germany) to 1 mL chloroform, 1 pellet of sodium hydroxide and dH2o at a total volume of 100 mL. The samples with unknown nitrite concentrations were injected into the FIA through the reaction manifold. Ammonium chloride was injected at a flow rate of 1.2 mL/min, and reacted with the sample in the reaction coil, which was 100 cm. Nitrite present in the sample reacts with a colouring reagent that consists of sulphanilamide and N-(1-naphthyl) ethylenediamine dihydrochloride, and this reaction forms diazonium salt, which causes a colour change that can be measured spectrophotometrically at 540 nm. The colour change was proportional to the concentration of nitrite, relative to the standard. The concentration of sodium nitrite was recorded as mg/kg, and the lower limit of reporting (LLOR) for sodium nitrite was 1 mg/kg.

### 2.3. Data Analysis

Statistical analysis was conducted using the IBM statistics package for social science (SPSS) version 25 (New York, NY, United States). Data was tested for normality using the Kolmogorov–Smirnov test. As carbohydrates, sugar, protein and salt were not normally distributed, they were logarithmically transformed. All other variables were normally distributed. The arithmetic mean (±SD) on their natural scale was used to present data. Differences in mean residual nitrite concentrations between smoked and unsmoked samples were determined using an independent t test, as were differences in manufacturer reported nutritional content. A one-way analysis of variance (ANOVA) test was used to assess differences in mean residual nitrite concentrations between different cuts of bacon—back, middle, streaky and medallion. An alpha value of <0.05 indicated significance, and least significant difference (LSD) was used to determine which groups were significantly different from each other. Pearson’s correlation coefficient was used to measure the linear correlation between residual nitrite, energy, fat, and protein content.

## 3. Results

Table 1 shows the descriptive statistics of *n* = 89 bacon samples collected from UK supermarkets. The mean (±SD) residual nitrite concentration of bacon samples collected from supermarkets was 10.80 mg/kg (±13.50), the limit of detection was 1 mg/kg, and 15 samples that were below this were imputed as 1 mg/kg. The range of residual nitrite concentrations for the 89 samples was 1–56 mg/kg. There was no difference in residual nitrite concentrations between smoked and unsmoked bacon (*p* = 0.691). As shown in Table 2, middle bacon had the highest residual nitrite concentration, and this was significantly higher than for the medallion (*p* = 0.008) and back (*p* = 0.027) bacon. Streaky bacon had the next highest residual nitrite concentration, and this was significantly higher than for the medallion (*p* = 0.013) and back (*p* = 0.005) bacon. Streaky bacon had the highest energy (268.33 kcal), and this was significantly higher than for back (*p* = 0.027), medallion (*p* ≤ 0.001), and diced (*p* = 0.030) bacon. Streaky bacon also had the highest fat levels (21.45 g), and this was significantly higher than for the back (*p* ≤ 0.001), medallion (*p* ≤ 0.001), and diced (*p* = 0.005) bacon. Streaky bacon also had the highest saturated fat content (8.68 g/100 g), and this was significantly higher than for the back (*p* ≤ 0.001), medallion (*p* ≤ 0.001), and diced (*p* = 0.009) bacon. Streaky bacon had the lowest protein content (18.00 g/100 g).

**Figure 1:**
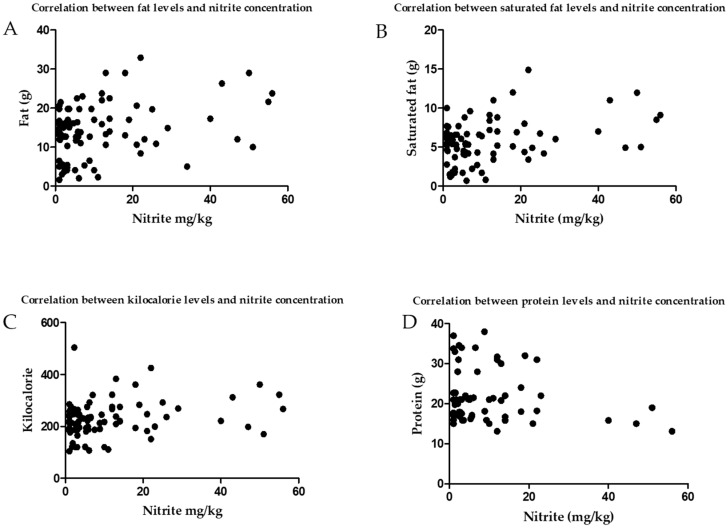
Correlation between residual nitrite concentration and (**A**) fat levels, (**B**) saturated fat levels, (**C**) kilocalorie levels, and (**D**) protein in bacon samples.

Conversely, medallions had the lowest residual nitrite (4.47 mg/kg), energy (149.90 kcal), fat (4.27 g), saturated fat (1.76 g/100 g), and the highest protein (23.56 g/100 g). Despite residual nitrite concentrations differing amongst the cuts, there was no difference in total salt concentrations between the four cuts. As shown in Figure 1, there was a positive correlation between residual nitrite and energy (*r* = 0.260, *p* = 0.022), fat (*r* = 0.263, *p* = 0.021), and saturated fat (*r* = 0.284, *p* = 0.012).

## 4. Discussion

We report the mean (±SD) residual nitrite concentration in bacon sold in UK supermarkets to be 10.80 (±13.50) mg/kg. Middle bacon had the highest mean residual nitrite level (26.00 ± 20.33 mg/kg), which was almost 3 times higher than back bacon, and more than 5 times higher than bacon medallions. Streaky bacon was next highest for residual nitrite levels (19.98 mg/kg), which was 4.5 times higher than bacon medallions. Streaky bacon had the highest energy, fat and saturated fat levels, and the lowest protein levels. Medallion bacon had the lowest energy, fat and saturated fat levels, and the highest protein levels. There was a correlation between the levels of residual nitrite and the levels of fat in all samples. There was no difference in residual nitrite concentrations between smoked and unsmoked bacon.

This was the first study in the UK to survey residual nitrite concentrations in bacon samples, and it was the most extensive worldwide, having the largest sample size. The results were similar to research surveying cured meats available in China and the USA. Yuan et al. [13] reported a mean residual nitrite concentration of ham as 16.1 mg/kg and sausage as 12.5 mg/kg. Their study collected 48 uncooked cured meat products, and the number of ham, sausage or other cured meats was not stated. A small study of nine uncooked bacon samples in the USA reported that the mean residual nitrite concentration was 10.43 mg/kg, which, despite the difference in sample size, is close to the value reported here [7]. Three further studies conducted in the United States reported mean residual nitrite values lower than the current study. A study of 20 uncooked, brine cured bacon samples collected from 5 cities in America found that the mean residual nitrite concentration was 6.8 mg/kg [14]. In a similarly designed USA based study, the mean residual nitrite concentration of bacon samples was 7.31 mg/kg [15]. Cassens (1997) [16] sampled three bacon samples and reported a mean residual nitrite concentration of 6.67 mg/kg. Conversely, studies conducted in Brazil, South Korea, and Australia reported levels over 2 times higher than that of this study. Twenty-one samples of Brazilian processed meat were analysed for residual nitrite concentrations and the mean value was reported as 22.4 mg/kg, and when stratifying the samples by type of processed meat, ham had an average value of 47.25 mg/kg and dry cured ham had a value of 9.4 mg/kg [17]. An in vitro study of bacterial growth on media treated with sodium chloride reported that a sample of bacon from South Korea had a residual nitrite concentration of 26 mg/kg, although the method of analysis was unclear [18]. Food Standards Australia and New Zealand (FSANZ) analysed 15 bacon samples and reported a mean of 26.6 mg/kg and a range of 12–45 mg/kg [19]. None of the aforementioned studies reported the cuts of bacon used. The most commonly consumed cut in the USA is streaky bacon, whilst the most commonly consumed in the UK is back bacon [20]. Our study highlights that streaky bacon in the UK tends to have higher residual nitrite concentrations than back bacon, and it may, therefore, be expected for residual nitrite levels in bacon from USA studies to be higher than is reported. The lower residual nitrite concentrations reported in the USA might be explained by the increased use of reductants in American-manufactured processed meat. Bacon produced in the USA must contain either ascorbate or sodium erythorbate, and these increase the reduction of nitrite to NO, making less nitrite available for quantification—this also subsequently makes less nitrite available for conversation to potentially carcinogenic nitrosamines. From the moment nitrite is added to a product, the amount of residual nitrite present begins to decline. The concentration of residual nitrite in sausages, has been observed to be between 5 and 19% of the original concentration at the use by date [21]. Neither ourselves or any of the previous studies can confidentially report the time between the addition of sodium nitrite and laboratory measurement of residual nitrite.

We report that middle bacon has the highest residual nitrite concentration and medallion has the lowest. There was a positive correlation between residual nitrite concentrations and fat levels, saturated fat, and energy. The higher residual nitrite levels in middle and streaky bacon are either due to more sodium nitrite being added during curing, or less incorporation of nitrite into the meat.

All samples in this study were well below the maximum permitted limit of 175 mg/kg specified by EFSA (2003) [11]. Concerns have been raised regarding the negative health effects that habitual consumption of nitrite may have. Nitrite has been shown to form NOCs in certain conditions, nitrosyl heme acts as a nitrosating agent for amines in this process, leading to potentially carcinogenic compounds [22]. A growing body of evidence has implicated nitrites in the development of CRC [23].

We are unable to definitively measure the total amount of nitrite added to bacon samples. When sodium nitrite is added to meat, it is reduced to NO and can no longer be detected. Furthermore, the rate of conversion is dependent on many factors, including pH, ratio of nitrite to myoglobin, temperature, water content, and the presence of reductants [24]. We, therefore, are unable to accurately calculate the exposure to nitrite adducts, which may enhance the risk of DNA damage.

Due to commercial availability (and consumer purchasing habits), this study had substantially more back bacon samples than any other cut. Future research could increase the quantity of middle, medallion, streaky, and diced bacon to allow for more accurate comparisons. Our study lacked other forms of nitrite-containing processed meat. Further research could include frankfurter, salami, ham, and pepperoni. Gaining an accurate estimation of the nitrite concentrations in all processed meat will allow for a better approximation of population exposure. The existing literature suggests that young children are at the greatest risk of exceeding the ADI for nitrite [25,26,27], even in Denmark where the maximum residual nitrite concentration allowed in bacon is 60 mg/kg. Further research on population exposure is needed to definitively draw conclusions from our results.

## 5. Conclusions

This study demonstrates that the mean residual nitrite concentration of bacon sold in the UK is similar to that sold in the US, and we also showed that the mean residual nitrite concentration is substantially lower than the allowable limit. Even samples with the highest measured concentrations are still considerably lower than this limit. It is not clear whether some manufacturers are adding sodium nitrite considerably below the allowable limit, or whether the sodium nitrite added is reacting with other constituents and is no longer measurable. Future research should investigate the kinetics of sodium nitrite reactions occurring after addition to meat. Given the current WHO position that sodium nitrite addition to processed meat is ‘probably carcinogenic’, it seems logical to add concentrations high enough to prevent spoilage but also sufficiently low enough to minimise health risk. Further research should be conducted to more precisely define an appropriate sodium nitrite concentration range.

## Figures and Tables

**Table 1:** Mean (SD) proximal analysis concentrations of bacon samples (*n* = 89) stratified by processing status.

	All Bacon	Smoked	Unsmoked	*p*
Residual nitrite mg/kg	10.80 (13.50)	10.44 (14.08)	11.03 (13.27)	0.691
Energy Kcal/100 g	224.68 (57.15)	229.83 (54.97)	221.63 (54.97)	0.556
Fat g/100 g	14.57 (6.20)	15.42 (6.69)	14.06 (5.91)	0.370
Saturated fat g/100 g	5.64 (2.58)	5.91 (2.83)	5.49 (2.43)	0.508
Carbohydrate g/100 g	0.46 (0.43)	0.53 (0.51)	0.44 (0.40)	0.146
Sugars g/100 g	0.35 (0.31)	0.39 (0.33)	0.33 (0.29)	0.342
Protein g/100 g	21.39 (6.47)	20.70 (6.10)	21.67 (6.66)	0.631
Salt g/100 g	3.12 (0.78)	3.04 (0.66)	3.28 (0.84)	0.202

An alpha value of <0.05 indicates significant difference, as determined by an independent T test. The difference is between smoked and unsmoked bacon. g, grams; Kcal, kilocalories; SD, standard deviation.

**Table 2:** Mean (SD) proximal analysis concentrations of bacon samples (*n* = 89) stratified by cut.

	Back (*n* = 52)	Streaky (*n* = 13)	Middle (*n* = 6)	Medallions (*n* = 15)	Diced (*n* = 3)
Residual nitrite mg/kg	8.87 (9.65) ¶,¥	19.87 (21.72) ‡,‖	26.00 (20.33) ‡,‖,ψ	4.47 (3.41) ¶,¥	6.37 (6.61) ¶,¥
Energy Kcal/100 g	229.56 (46.19) ¶,‖	268.33 (60.04) ‡,‖,ψ	260.00 (56.67) ‖	149.90 (44.21) ‡,¶,¥	197.67 (18.48) ¶
Fat g/100 g	15.07 (4.58) ¶,‖	21.45 (5.78) ‡,‖,ψ	18.26 (3.67) ‖	4.27 (1.56) ‡,¶,‖,ψ	13.13 (0.75) ¶,‖
Saturated fat g/100 g	5.70 (2.00) ¶,‖	8.68 (2.48) ‡,‖,ψ	7.44 (0.97) ‖	1.76 (0.69) ‡,¶,¥,ψ	5.33 (0.12) ¶,‖
Carbohydrate g/100 g	0.54 (0.45)	0.24 (0.34)	0.63 (2.87)	0.23 (0.32)	0.50 (0.71)
Sugars g/100 g	0.39 (0.32)	0.22 (0.22)	0.38 (0.25)	0.25 (0.25)	0.50 (0.71)
Protein g/100 g	21.76 (6.70)	18.00 (5.68)	21.33 (7.22)	23.56 (6.37)	19.27 (2.37)
Salt g/100 g	3.10 (0.70)	3.10 (0.56)	3.30 (0.94)	3.71 (1.16)	3.60 (1.21)

An alpha value of <0.05 indicates significant difference, as determined by a one-way ANOVA comparing cuts of bacon. g, grams; Kcal, kilocalories; SD, standard deviation. Groups significantly different from back are indicated by ‡, groups significantly different from streaky are indicated by ¶, groups significantly different from middle are indicated by ¥, groups significantly different from medallion are indicated by ‖, and groups significantly different from diced are indicated by ψ.
